# Editorial: Nanotechnology for Antimicrobials

**DOI:** 10.3389/fmicb.2020.01421

**Published:** 2020-07-09

**Authors:** Renata K. T. Kobayashi, Gerson Nakazato

**Affiliations:** Department of Microbiology, Center of Biological Sciences, State University of Londrina, Londrina, Brazil

**Keywords:** nanoparticles, drug delivery, microbial pathogens, therapy, disease prevention, synthesis, resistance

Nanotechnology has presented many advantages and benefits in science and industrial process. Nanoparticles have special characteristics that optimize the biological, physical, and chemistry properties being studied for many aims. The use of nanoparticles resulted in great evidences in Microbiology including antimicrobial activity against bacteria, fungi, virus, and protozoa. In this Research Topic, different nanoparticles and their strategies showed efficient antimicrobial effects with potential applications to control infections and biological contaminations. All articles showed the antimicrobial activity of these nanoparticles, direct or indirectly, against pathogenic microorganisms that cause severe infections, including those multidrug-resistant to the conventional antimicrobials.

The global spread of multidrug-resistant (MDR) microbial pathogens is currently considered one of the principal threats to global public health according to the World Health Organization (WHO) and it is estimated that unless action is taken, the number of deaths worldwide could increase to 10 million each year by 2050. Although the emergence of antimicrobial resistant bacteria is a natural process, this process can be accelerated by the non-rational use of antimicrobials, inadequate surveillance, and the insufficiently controlled regulation of antibiotics use in clinical medicine and in the livestock industry.

In view of the emergence of MDR microorganisms associated with scarcity in the discovery of new antibiotics, new anti-infectious strategies, need to be increasingly explored. In this Research Topic new effective strategies are showed as: metallic, biominerals and selenium nanoparticles with antimicrobial action (Basera et al.; Gunti et al.; Lakshmeesha et al.; Masum et al.; Nagaraj and Samiappan; Santos et al.), metallic nanoparticles that inhibited the biofilms formation (Fonseca et al.), magnetic nanoconjugate antibiotics that improved the antimicrobial action (Armenia et al.; Chen et al.), prebiotics in nanoparticles to better the antibacterial properties of probiotics (Hong et al.), antimicrobial peptides and bacteriophages nanoencapsulated in liposomes for prolonged delivery (Cinquerrui et al.; Zambom et al.).

All articles in this Research Topic proved the antimicrobial action of the respective compounds against pathogenic bacteria or fungi and some of them were effective against *Clostridium difficile*, Methicillin-resistant *Staphylococcus aureus* (MRSA) and Vancomycin-resistant *Enterococcus* (VRE) that are part of the threat list considered the most alarming antibiotic-resistant microorganisms according to the Centers for Disease Control and Prevention (CDC).

Nagaraj and Samiappan performed the biomimetization of Hydroxyapatite (HA) with *Azadirachta indica* (AI) and the preparation of HA-AI composite which showed potent antibacterial activity against *Staphylococcus aureus* ATCC 700699, which strain is Methicillin resistant (MRSA), resistant to Oxacillin and shows reduced Vancomycin susceptibility. Armenia et al. also tested magnetic nanoconjugated teicoplanin (NP-TEICO) against MRSA and Vancomycin-resistant *Enterococcus* (VRE). Both MRSA and VRE are classified as serious threat according to CDC, since they are resistant to the antimicrobials usually prescribed to combat these species, which can make it difficult to treat serious infections in hospitalized or community patients, highlighting the importance of developing new antimicrobials against these pathogens.

Chen et al. developed a sporicidal and antimicrobial vancomycin-loaded spore targeting iron oxide nanoparticle (van-IONP) that selectively binds to *C. difficile* spores. This microorganism is classified as urgent threat by CDC once it is a major cause of healthcare-acquired life-threatening diarrhea associated with the rising use of antibiotics, which is responsible to substantial mortality around the world. Van-IONP can target and completely cover spore surfaces. They not only successfully delayed the germination of the spores but also inhibited of vegetative cell outgrowth. This delivery therapy showed advantages over traditional therapeutics in treating *C. difficile* infection.

The articles published in this Research Topic approached some specific aspects about antimicrobial activity of nanoparticles in different fields (human medicine, veterinary, and foods). Nagaraj and Samiappan showed that the biomimetization of hydroxyapatite has an interesting anti-inflammatory potential property over lipopolysaccharide (bacterial endotoxin from Gram-negative) besides the antibacterial activity with a potential application in dentistry and orthopedic. Often inflammatory reactions are very important for treatment of infections because in this case the lipopolysaccharide causes septic shock in patients, leading to death.

Drug delivery strategy has been studied to optimize the antimicrobial activity using different carriers and actives. Many drug delivery models are similar to cancer treatment including the use of nanocarriers. Zambom et al. performed a drug delivery strategy using nanoliposomes with Histatin 5 inhibiting the growth of *Candida albicans*. Already Cinquerrui et al. used nanoliposomes encapsulated phages like a “Trojan Horse.” Nanocarriers conjugated with antibiotics using magnetic nanoparticles were also suggested by Armenia et al. as potential application to infections sites.

Syntheses of different types of nanoparticles were also approached in some studies of this Research Topic. The biological synthesis was well-performed in some articles resulting in metallic nanoparticles with antimicrobial properties. Lakshmeesha et al. showed that the zinc oxide nanoparticles biosynthesized using a flower bud extract reduced the production of mycotoxins of *Fusarium graminearum* suggesting the use of these nanoparticles as a potential antifungal in agriculture and food industry. The pythofabrication of selenium nanoparticles using fruit extract was performed by Gunti et al. and these nanoparticles showed antimicrobial, antioxidant, and biocompatibility properties. Biogenic silver nanoparticles have been an interesting alternative as antibacterial as demonstrated (Basera et al.). Santos et al. also got great results using biogenic nanosilver in the treatment of *Corynebacterium pseudotuberculosis* infections in small ruminants. In this last study, the results *in vivo* indicate that biogenic nanosilver can be applied as an alternative in the treatment of infections in Veterinary field. These studies revealed that the antimicrobial properties of biogenic nanoparticles depend on the source of reducing agent as well as active molecules.

Some bacterial strains are able to protect themselves against antimicrobials by resistance mechanisms or bacterial structure. Spore forming is a classic example of bacterial structure of resistance. Still, the sporicidal effect of vancomycin-loaded nanoparticles against *Clostridium* was demonstrated by Chen et al. in this Research Topic. Masum et al. using biogenic silver nanoparticles managed to disturb biofilm formation and swarming ability. Another study about biofilm inhibition was performed by Fonseca et al., in this case using nanocomposite of silver-doped ZnO and AgO nanocrystals to control biofilm formation in eggshell. Structural and inhibition biofilms have been studied in Microbiology and Pathogenesis mainly for some materials such as medical devices. These biofilms are difficult to eliminate or remove in the surfaces. In this case, these nanoparticles prevented the biofilm formation suggesting a potential use in materials.

Another way to control infections is the probiotics, prebiotics and symbiotics including the application of nanoparticles. Hong et al. described pullulan nanoparticles as prebiotics enhancing the antibacterial effect of *Lactobacillus* against *Listeria monocytogenes*. This study indicates that some nanoparticles can act on microbiome interacting positively with other bacteria on prevent of infections.

The results of these studies demonstrated the importance of Nanotechnology for infection control in different field even as model studies for antimicrobial activity. The nanoparticles synthesized biologically (“green nanoparticles”) and biocompatible present in some articles corroborate with the concerns about safety and ecology in the use of these antimicrobials. Many applications and products using nanoparticles can be generated from this Research Topic.

Future perspectives revolve around new strategies and development of products to prevent, control and treat microbial infections in humans and other animals, including viral infection, as seen in the actual pandemic scenario.

## Author Contributions

All authors listed have made a substantial, direct and intellectual contribution to the work, and approved it for publication.

## Conflict of Interest

The authors declare that the research was conducted in the absence of any commercial or financial relationships that could be construed as a potential conflict of interest.

